# Chronotype and Cancer: Emerging Relation Between Chrononutrition and Oncology from Human Studies

**DOI:** 10.3390/nu17030529

**Published:** 2025-01-31

**Authors:** Justyna Godos, Walter Currenti, Raffaele Ferri, Giuseppe Lanza, Filippo Caraci, Evelyn Frias-Toral, Monica Guglielmetti, Cinzia Ferraris, Vivian Lipari, Stefanía Carvajal Altamiranda, Fabio Galvano, Sabrina Castellano, Giuseppe Grosso

**Affiliations:** 1 Department of Biomedical and Biotechnological Sciences, University of Catania, 95123 Catania, Italy; justyna.godos@unict.it (J.G.);; 2 Center for Human Nutrition and Mediterranean Foods (NUTREA), University of Catania, 95123 Catania, Italy; 3 Oasi Research Institute-IRCCS, 94018 Troina, Italy; 4 Department of Surgery and Medical-Surgical Specialties, University of Catania, 95125 Catania, Italy; 5 Department of Drug and Health Sciences, University of Catania, 95125 Catania, Italy; 6 School of Medicine, Universidad Espíritu Santo, Samborondón 0901952, Ecuador; 7 Human Nutrition and Eating Disorder Research Center, Department of Public Health, Experimental and Forensic Medicine, University of Pavia, 27100 Pavia, Italy; 8 Laboratory of Food Education and Sport Nutrition, Department of Public Health, Experimental and Forensic Medicine, University of Pavia, 27100 Pavia, Italy; 9 Research Group on Food, Nutritional Biochemistry and Health, Universidad Europea del Atlántico, Isabel Torres 21, 39011 Santander, Spain; 10 Universidad de La Romana, La Romana 22000, Dominican Republic; 11 Universidad Internacional Iberoamericana, Campeche 24560, Mexico; 12 Universidade Internacional do Cuanza, Cuito EN250, Angola; 13 Fundación Universitaria Internacional de Colombia, Bogotá 111321, Colombia; 14 Department of Educational Sciences, University of Catania, 95124 Catania, Italy

**Keywords:** chronotype, sleep, time-restricted eating, circadian rhythm, metabolic dysregulation, gut microbiota, cancer

## Abstract

Fasting–feeding timing is a crucial pattern implicated in the regulation of daily circadian rhythms. The interplay between sleep and meal timing underscores the importance of maintaining circadian alignment in order to avoid creating a metabolic environment conducive to carcinogenesis following the molecular and systemic disruption of metabolic performance and immune function. The chronicity of such a condition may support the initiation and progression of cancer through a variety of mechanisms, including increased oxidative stress, immune suppression, and the activation of proliferative signaling pathways. This review aims to summarize current evidence from human studies and provide an overview of the potential mechanisms underscoring the role of chrononutrition (including time-restricted eating) on cancer risk. Current evidence shows that the morning chronotype, suggesting an alignment between physiological circadian rhythms and eating timing, is associated with a lower risk of cancer. Also, early time-restricted eating and prolonged nighttime fasting were also associated with a lower risk of cancer. The current evidence suggests that the chronotype influences cancer risk through cell cycle regulation, the modulation of metabolic pathways and inflammation, and gut microbiota fluctuations. In conclusion, although there are no clear guidelines on this matter, emerging evidence supports the hypothesis that the role of time-related eating (i.e., time/calorie-restricted feeding and intermittent/periodic fasting) could potentially lead to a reduced risk of cancer.

## 1. Introduction

Circadian rhythms, the internal biological clocks that align physiological and behavioral processes with the 24 h light–dark cycle, are fundamental to maintaining health and homeostasis in humans [1]. These rhythms orchestrate a vast array of functions, including the regulation of sleep–wake cycles, hormonal secretion, metabolism, immune responses, and cellular repair mechanisms [2]. At the heart of this system lies the suprachiasmatic nucleus, a master clock located in the hypothalamus [3]. The suprachiasmatic nucleus coordinates peripheral clocks present in nearly all tissues and organs, such as the liver, pancreas, and adipose tissue, ensuring that internal processes remain synchronized with environmental cues, particularly light [4]. By maintaining this harmony, circadian rhythms enable organisms to anticipate daily changes in their environment and optimize physiological processes. However, disruptions to these rhythms, commonly referred to as circadian misalignment, can have profound effects on health, contributing to an increased risk of chronic diseases, including cancer [5]. The connection between circadian rhythms and cancer has garnered significant attention in recent years. DNA repair, cell cycle regulation, and apoptosis are all under circadian control, and the disruption of these processes may lead to genomic instability, the accumulation of mutations, and unregulated cell growth [6]. However, the underlying mechanisms by which circadian disruption influences cancer risk remain an active area of research. While some studies suggest that clock gene dysfunction directly contributes to tumorigenesis, others propose that the associated metabolic and hormonal disturbances, such as insulin resistance, chronic inflammation, and altered melatonin secretion, may mediate this relationship [7].

Circadian misalignment, caused by factors such as shift work, irregular sleep schedules, and prolonged exposure to artificial light, can interfere with all the aforementioned cellular processes that are crucial for preventing malignancy. Emerging evidence shows that diet is strongly related to sleep [8,9,10,11], representing one of the most important determinants of sleep quality or, conversely, circadian misalignment. In this context, chrononutrition has emerged as a promising field that examines the interaction between diet, eating behaviors, and circadian rhythms [12]. Chrononutrition focuses not only on the nutritional content of meals but also on the timing, frequency, and regularity of eating patterns [13]. Only recently has such a behavior been considered a genetically predetermined phenotype known as “chronotype” [14]. These time-related behaviors underlying that when food is consumed is as important as what is consumed, given the interplay between circadian clocks and metabolic pathways. Since feeding and fasting cycles are key regulators of peripheral clocks in metabolic tissues, such as the liver and adipose tissue, the disruption of these cycles through irregular meal timing or late-night eating can lead to metabolic dysregulation and may influence cancer risk [15]. This misalignment may be mediated by a desynchronization between central and peripheral clocks, impairing the expression of clock-regulated genes involved in metabolic and inflammatory pathways. Either way, there is an ongoing discussion on whether chrononutrition may play a role in cancer risk and through which pathways. Additionally, evidence up to date mostly relies on pre-clinical mechanistic studies, while data from humans have only been published recently and are not included in the only existing summary of literature accounting for chronotype and human health [16]. The aim of this review is to summarize current evidence from epidemiological studies and provide an overview of the hypothesized mechanisms underlying an association between chronotype, chrononutrition, and cancer.

## 2. Chronotype and Time-Restricted Eating (TRE)

Circadian rhythms at the genetic level are regulated by a network of “clock genes”, which function within a feedback mechanism to generate 24 h oscillations in gene expression [17]. Important clock genes, including CLOCK, BMAL1, PER, and CRY, interact through a series of transcriptional and translational feedback loops that orchestrate rhythmic cellular activities [18]. The molecular cycle begins with the activation of CLOCK and BMAL1, which together form a complex that drives the transcription of PER and CRY genes: as PER and CRY proteins accumulate, they suppress the CLOCK–BMAL1 complex, creating a time delay that facilitates the generation of a 24 h rhythm [19]. Eventually, these proteins degrade, lifting the inhibition and restarting the cycle. The regulation of clock proteins also involves post-translational modifications, including phosphorylation, O-GlcNAcylation, and acetylation, which determine their stability or degradation [19]. This feedback loop is a core component of the circadian system and is highly conserved across a wide range of species, from lower organisms to humans, underscoring its evolutionary significance in regulating circadian processes.

Chronotype refers to an individual’s natural preference for the timing of sleep, wakefulness, and associated behavioral and physiological activities within a 24 h cycle [20]. Chronotype varies significantly across populations, with individuals classified along a spectrum from “morning types” (early risers) to “evening types” (late risers), with “intermediate types” falling in between [21]. This intrinsic temporal preference is being recently studied for impacting not only sleep patterns but also cognitive performance, metabolic health, and susceptibility to various diseases [22,23]. Importantly, chronotype is influenced by a combination of both genetic and environmental factors. Specific polymorphisms in genes such as *PER3*, *CLOCK*, and *CRY1* have been associated with morningness or eveningness tendencies [22,23]. Also, environmental factors, particularly light exposure, may influence chronotype. For instance, individuals exposed to artificial light in the evening, such as blue light from screens, seasonal variations, and geographical factors such as altitude/longitude, can delay melatonin secretion and shift individuals toward an evening chronotype [24]. However, whether a misalignment between a genetic predisposition and an opposite environmental stimulus could be considered detrimental to health is still an unexplored hypothesis.

In this context, chronotype may predict the timing of the day dedicated to eating. Chrononutrition has emerged as an interesting field of research focusing on aspects of the diet other than its content, including (i) irregularity (i.e., inconsistency or inconsistent meal routine), (ii) frequency (the number of meals or eating occasions daily), and (iii) clock time (actual time of intake), for example, breakfast skipping and consuming meals late at night and vice versa [25]. All these features may characterize meal patterns comprehensively defined as time-restricted eating (TRE), also referred to as time-restricted feeding (TRF) in animal studies, in which food intake is limited to a specific window of time (typically ranging from 4 to 12 h), ideally during the daylight hours (“early” TRE, in contrast to “late” TRE, in which food intake occurs during evening time) [26].

There is a dual relation between diet and sleep, with worse diet quality leading to poor sleep quality [8,27,28] and decreased sleep duration associated with higher dietary intakes [29,30]. The resulting effects lead to a misalignment of dietary and sleeping habits with internal circadian clocks (molecular, cellular, and systemic), leading to potential risks to health [31]. In contrast, when the eating time comprises the early hours of the day, the food intake aligns with the active phase of the circadian cycle and results in the maintenance of the physiological functioning of the human body [32].

## 3. Evidence from Human Studies on Chrononutrition and Cancer

### 3.1. Chronotype and Cancer

Epidemiological studies on chronotype support the hypothesis of a potential association with several health conditions, especially cardio-metabolic conditions [16]; however, data on cancer are scattered and only more recent. In fact, previous studies have not been conducted on chronotype per se but on surrogate information, such as shift working. Occupational demands, commonly referred to as “professional jet lag,” can misalign biological rhythms with societal schedules, exacerbating chronotype discrepancies and leading to adverse health outcomes [33]. The rationale to use a working schedule as a proxy for chronotype lays the ground on the hypothesis that individuals with an evening chronotype would be more likely to choose a job implying working at night or having night shifts. Some comprehensive analyses of the literature showed that shift workers have a higher risk of breast [34] and prostate tumors [35], and inconsistent results concerning colorectal and other types of cancers [36,37], suggesting that an evening chronotype could be a detrimental trait concerning cancer risk.

More recent studies started investigating chronotype per se as a risk factor for cancer (Table 1). Chronotype is generally identified by asking specific questions on personal perception of preference for doing any activity or validated instruments [38]. An investigation conducted on members of the California Teachers Study cohort including 437 endometrial cancer cases and 26,753 cancer-free controls showed that women who were definite evening types had statistically significantly increased odds of having cancer (OR = 1.44, 95% CI 1.09–1.91) compared with morning types [39]. However, a case–control study of 180 incident cases of endometrial cancer and 218 hospital controls reported inconsistent patterns for chronotype [40]. Another study from the California Teachers Study cohort including 2719 cases of primary invasive breast cancer and 36,967 participants showed a higher likelihood for evening compared with morning chronotypes and having cancer (OR = 1.20, 95% CI: 1.06–1.35) [41]. Another report longitudinally assessing the relation between chronotypes and breast cancer risk including 1085 incident cancer cases in 38,470 cancer-free participants from 2012 through 2019 showed that the risk for evening chronotypes compared with morning chronotypes was somewhat elevated (HR = 1.19, 95% CI 1.04–1.36) [42]. These findings are similar to those from another prospective study conducted in the context of the Alberta’s Tomorrow Project cohort following a subset of participants (*n* = 19,822) reporting 1322 incident cancer cases in the group for which information on sleep timing analyses were available and showed that a later sleep timing midpoint indicative of an evening chronotype versus an intermediate sleep timing midpoint was associated with an increased incidence of combined (HR = 1.20, 95% CI: 1.04–1.37) and breast (HR = 1.49, 95% CI: 1.09–2.03) cancers [43]. Also, a study from the UK Biobank including 216,702 participants and resulting in 2367 incident breast cancer showed that an evening chronotype was associated with breast cancer risk in females who carry the rs10830963 G risk allele in melatonin receptor 1B [44]. However, in a study from the Nurses’ Health Study II conducted on 72,517 women, participants who self-reported as neither morning nor evening types had a 27% increased risk of breast cancer (OR = 1.27, 95% CI: 1.04–1.56) compared with definite morning types, while none of the other chronotypes were significantly associated with breast cancer risk [45]. Also, an early nested case–control study including 218 cases of breast cancer and 899 age-matched controls from military employees in Denmark explored the role of shift working by also taking into account the individual preference for morning or evening activities, resulting in an increased risk of breast cancer in shift workers, especially if they self-identified as morning chronotypes (OR = 3.9, 95% CI: 1.6–9.5) [46]. An earlier report from the UK Biobank conducted on 469,691 individuals free of lung cancer at recruitment and 2177 incident lung cancer cases reported that an evening preference was associated with an elevated lung cancer risk compared with a morning preference (HR = 1.25, 95% CI: 1.07–1.46) [47]. In an updated report from the same cohort including 382,966 participants and 3664 incident lung cancer, the analysis excluding participants reporting shift work at baseline showed that slight and definite evening chronotypes were at a greater risk of lung cancer compared with the definite morning chronotype (HR = 1.17, 95% CI, 1.06–1.28 and HR = 1.37, 95% CI, 1.21–1.54, respectively) [48]. However, a smaller study with a case–control design showed uncertainty in the association between chronotype and lung cancer, reporting null results [49]. A study from the Older Finnish Twin Cohort including 11,370 twins and 602 incident cases and 110 deaths from prostate cancer showed that “somewhat evening” types had a significantly increased risk of prostate cancer (HR = 1.3, 95% CI: 1.1–1.6) compared with “definite morning” types [50]. Also, a previous case–control study including 819 incident prostate cancer cases and 879 controls frequency matched by age reported an increased prostate cancer risk among men with an evening chronotype (OR = 1.96, 95% CI: 1.04–3.70) [51]. However, data from the UK Biobank on 213,999 individuals free of cancer at recruitment accounting for 6747 incident cases reported that chronotype was not associated with cancer risk [52]. The reported results are inconsistent and potentially biased by shift working in other studies as well, since a case–control study including 465 prostate cancer cases and 410 controls showed that individuals had an increased likelihood to have cancer if they were shift workers, especially for rotating night shifts, with a marginal association with chronotype [53]. Similarly, another study conducted on 1095 prostate cancer cases and 1388 randomly selected population controls reported a higher risk of tumors, particularly among subjects with longer durations of shift work, with more pronounced risks among subjects with an evening chronotype, as well as increased risks in morning chronotypes after long-term night work [54]. In another study from the UK Biobank involving 393,114 participants and documenting 294 incident esophageal adenocarcinoma and 95 squamous cell carcinoma cases, the evening chronotype was associated with an elevated risk of cancer diagnosed after 2 years of enrollment (HR = 2.79, 95% CI: 1.32–5.88) [55]. In contrast, a population-based case–control study with 496 epithelial ovarian cancer cases and 906 controls reported a non-significant association between being shift workers and cancer, although it was more pronounced among women who self-identified as having a “morning” chronotype [56]. Also, no associations were found for the evening chronotype and risk of pancreatic cancer in 475,286 participants from the UK Biobank (“definitely” an evening person versus “definitely” a morning person, HR = 0.99, 95% CI: 0.77–1.29) [57].

Some of these associations have also been supported by results from Mendelian randomization studies (Table 2), which is an analytical method used in epidemiology and genetics to infer causal relationships between modifiable risk factors (exposures) and health outcomes using genetic variants associated with the specific exposures of interest [58]. It offers a more robust way to understand how these exposures affect outcomes, as germline genetic variants are randomly inherited from parents, making them unlikely to be associated with confounding factors that could influence the relationship between exposure and outcome. As a result, genetic variants serve as a tool to link a risk factor with an outcome, enabling an estimation of the effect with reduced confounding and bias compared with traditional epidemiological methods [59]. A study using data from the UK Biobank that comprehensively assessed several cancer sites showed a positive association between the definite evening chronotype and the incidence risk of overall cancer, breast cancer, lung cancer, endometrial cancer, and ovarian cancer, and a causal relationship from the Mendelian randomization analysis showed a protective effect of the definite morning chronotype on the risk of overall cancer (OR = 0.91, 95% CI: 0.85–0.97 per category increase), lung cancer (OR = 0.34, 95%CI: 0.26–0.44 per category increase), and breast cancer (OR = 0.69, 95% CI: 0.59–0.80 per category increase), as well as slightly weaker effects for ovarian cancer (OR = 0.61, 95% CI: 0.39–0.97 per category increase) and endometrial cancer (OR = 0.62, 95%CI 0.43–0.91 per category increase), with positive associations between the definite evening chronotype and these types of cancers confirmed as well [60]. Similarly, another Mendelian randomization study including 133,384 breast cancer cases confirmed that daytime dozing and a genetically determined morning chronotype are causally linked to a lower risk of breast cancer [61]. A study including 133,384 breast cancer cases from the Breast Cancer Association Consortium (BCAC) performed a cross-trait meta-analysis identifying 78 loci shared between chronotype and breast cancer, while the Mendelian randomization demonstrated a significantly reduced risk of overall breast cancer (OR = 0.89, 95% CI: 0.83–0.94) for the genetically predicted morning chronotype [62]. Another study showed that a genetic liability to the morning chronotype was associated with a reduced risk of overall digestive tract cancer (OR = 0.94, 95% CI: 0.90–0.98), stomach cancer (OR = 0.84, 95% CI: 0.73–0.97) and colorectal cancer (OR = 0.92, 95% CI: 0.87–0.98) in the UK Biobank (11,952 cases) and FinnGen (7638 cases) studies [63]. In contrast, another study conducted on similar datasets but specific to the causal association between sleep traits and colon and rectum cancers showed that the morning chronotype was marginally associated with the risk of colon cancer (OR = 1.004, 95% CI: 1.000–1.007) [64]. Another Mendelian randomization study including GWASs data from the UK Biobank and FinnGen reported a potential causal relation between thyroid cancer and the genetically determined morning chronotype [65]. A study from the Prostate Cancer Association Group to Investigate Cancer-Associated Alterations in the Genome (PRACTICAL) Consortium comprising 79,148 cases reported that individuals with genetically predicted morningness had a reduced causal risk of prostate cancer (OR = 0.71, 95% CI: 0.54–0.94) compared with those with eveningness [66]. In contrast, a study testing if comprehensively chronotype, getting up in the morning, sleep duration, nap during the day, or sleeplessness was causally associated with the risk of lung cancer in two large consortia (UK Biobank and International Lung Cancer Consortium) showed that sleeplessness was associated with a higher risk of lung adenocarcinoma while sleep duration played a protective role in lung cancer, with no significant associations with chronotype per se [67].

### 3.2. Time-Restricted Eating and Cancer

Studies specifically conducted on the association between TRE and cancer risk are relatively scarce [68]. Some information on this matter can be drawn from studies conducted on exposures implying habits related to TRE (e.g., nighttime fasting, breakfast skipping, and meal timing; Table 3). There is compelling evidence showing a relation between general sleep duration (either long or short sleep) and increased risks of breast [69], lung [70], and colorectal cancers [36], as well as cancer-specific mortality [71], but not on prostate cancer risk [72] (although with no mention to eating habits). Among more specific studies, a study from a Spanish multicase–control (MCC) study (2008–2013) recruiting individuals who never had night shift work including 607 prostate cancer cases and 848 population-based controls showed that fasting for more than 11 h overnight was associated with a reduced risk of prostate cancer compared with those fasting for 11 h or less (OR = 0.77, 95% 0.54–1.07) [73]. Moreover, participants reporting a combination of long nighttime fasting and early breakfast were less likely to have prostate cancer compared with those reporting a short nighttime fasting and a late breakfast [73]. However, in another study from the same case–control framework including 1181 breast cancer cases and 1326 population controls, the same association between nighttime fasting duration and cancer risk was not observed, although in premenopausal women, each hour later in breakfast time was associated with an 18% increase in breast cancer risk (OR = 1.18, 95% CI: 1.01–1.40) [74]. Another study examining baseline data from 159 breast cancer patients in comparison with a national survey of Australian women revealed that instances of eating after 8 PM, short nightly fasting duration (<13 h), and long sleep duration (>9 h/day) were higher in breast cancer survivors than women in the national survey [75]. However, evidence from other studies makes it unclear whether the relation with cancer risk relies on a long fasting period or the alignment of eating occasions with circadian cycles. In fact, breakfast skipping has been associated with a higher risk of cancer mortality based on comprehensive evidence from the scientific literature [76]. Moreover, breakfast skipping, more than prolonged nighttime fasting, is likely associated with a pro-inflammatory and dysmetabolic profile [77]. Although specific evidence on cancer is limited, a study conducted on the NutriNet-Santé, a large cohort of 41,389 day-working French adults followed up to count 1732 first primary incident cancer cases, showed that the risk of breast (HR = 1.48, 95% CI: 1.02–2.17) and prostate cancer (HR = 2.20, 95% CI: 1.28–3.78) was increased in late eaters (last eating episode after 9:30 pm), but no associations were observed between cancer risk and number of eating episodes, nighttime fasting duration, and time of first eating episode [78].

## 4. Mechanisms Underlying the Role of Chronotype and Chrononutrition on Cancer

The mechanisms linking chronotype, chrononutrition, and cancer range from genetic mechanistic factors to adverse metabolic outcomes.

### 4.1. Cell Cycle Regulation, Chronotype and Cancer

**Figure 1:**
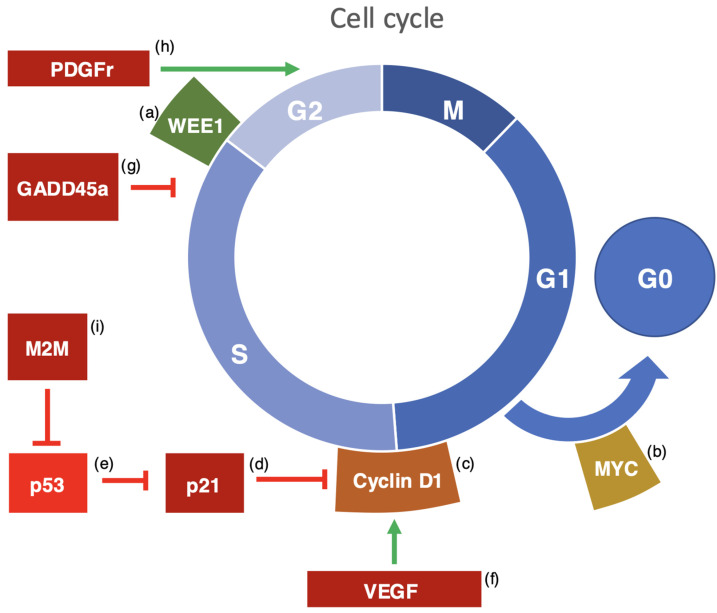
Major genes demonstrating circadian expression potentially involved in cancer biology. (a) WEE1 is an important kinase involved in controlling the G2/M transition of the cell cycle. It works by adding phosphate groups to CDK1 (Cyclin-Dependent Kinase 1), which inhibits its activity and prevents the cell from entering mitosis too early. When WEE1 expression is disrupted, it can lead to abnormal cell cycle progression and increased tumor cell growth, especially in both blood-related and solid cancers. The circadian regulation of WEE1 ensures that cell cycle checkpoints function correctly at various times of the day, helping to prevent errors in DNA replication that could result in cancer. Disruptions in WEE1 expression in tumors can cause uncontrolled cell division and genomic instability. (b) The MYC proto-oncogene produces transcription factors that regulate the transition from the G0 to G1 phase of the cell cycle. As a key regulator of cell growth and division, MYC is often overexpressed in many types of cancer. Excessive MYC activity promotes cell proliferation by activating genes that drive the cell cycle forward. The circadian regulation of MYC helps balance cell growth and division, but when this regulation is disrupted, it can lead to uncontrolled cell division, a hallmark of cancer. (c) Cyclin D1 is crucial for the G1/S transition in the cell cycle, where it activates CDK4 and CDK6, leading to the phosphorylation of the retinoblastoma protein and promoting cell cycle progression. Cyclin D1 is often overexpressed in various cancers, which accelerates entry into the S phase and boosts cell proliferation. Its circadian regulation ensures the proper timing of its expression during the cell cycle, but when circadian rhythms are disrupted, Cyclin D1 regulation is lost, which can contribute to cancer progression. (d) CDKN1A, or p21, is a cyclin-dependent kinase inhibitor that regulates the cell cycle by preventing cyclin–CDK complexes from functioning. As a tumor suppressor, p21 helps arrest the cell cycle, but its expression can be dysregulated in different cancers. In some cancers, p21 is overproduced, which may help cells resist apoptosis, while in others, its expression is too low, leading to unchecked cell growth. The circadian expression of p21 ensures the proper regulation of the cell cycle, but the disruption of circadian rhythms can impair this regulation, potentially driving tumor formation. (e) p53 is a tumor suppressor gene essential for maintaining genomic stability by controlling cell cycle arrest, DNA repair, and apoptosis. Disruptions to the circadian rhythm can impair p53′s function, increasing the risk of genomic instability and cancer. Research shows that altered circadian rhythms reduce p53 activity, preventing the cell from halting the cycle in response to DNA damage, allowing for continued proliferation despite genetic mutations. The loss of p53 function is a key factor in many cancers, and circadian disruptions can worsen this issue, elevating the likelihood of cancer development. (f) Vascular Endothelial Growth Factor (VEGF) regulates normal cell growth and angiogenesis, the formation of new blood vessels. In cancer, VEGF promotes the growth of blood vessels that supply tumors with oxygen and nutrients, supporting tumor expansion. Circadian rhythms influence VEGF expression, and when circadian timing is disrupted, VEGF levels can rise, enhancing angiogenesis and tumor metastasis. (g) Growth Arrest and DNA Damage-Inducible Protein alpha (GADD45a) plays a role in responding to cellular stress and DNA damage. It helps repair DNA and control the cell cycle by interacting with cdc2/cyclinB1 kinases to inhibit cell cycle progression during the G2/M and S phases. GADD45a is often underexpressed in cancers, and its circadian regulation ensures it is available to repair DNA damage when needed. Disruptions in GADD45a expression due to circadian misalignment can prevent effective DNA repair, leading to genomic instability and promoting cancer progression. (h) PDGF Receptor (PDGFr) is a receptor tyrosine kinase involved in cellular growth, survival, and migration. PDGFr plays a role in cancer progression by promoting tumor cell proliferation, migration, and invasion. The dysregulation of PDGF receptor signaling has been linked to various cancer types, and circadian disruption can alter its expression, enhancing tumor growth and metastasis. (i) MDM2 is a negative regulator of p53, forming a feedback loop that controls p53 levels by promoting its degradation. In healthy cells, MDM2 prevents the accumulation of excess p53, ensuring proper cell cycle control. However, MDM2 is often overexpressed in cancers, leading to reduced p53 activity and the evasion of cell cycle arrest and apoptosis. The circadian regulation of MDM2 ensures this feedback loop works as intended, but the disruption of circadian rhythms can prevent proper p53 regulation, allowing damaged cells to proliferate and contribute to cancer development.

Several mechanisms have been proposed to explain how circadian disruption can promote tumorigenesis through genetic pathways. One key pathway involves circadian rhythms’ impact on cell cycle regulation. Evolutionarily, circadian oscillations in gene expression may have evolved as a response to environmental factors like UV light exposure or periods of increased cellular replication. During these times, DNA damage byproducts can accumulate, impairing DNA stability and gene expression, which increases the risk of uncontrolled cell proliferation and reduced apoptosis, thus fostering cancer development. Certain clock-controlled genes, including those encoding cell cycle regulators and DNA damage response proteins, exhibit circadian expression patterns, which, when disrupted, can lead to uncontrolled cell division and the evasion of programmed cell death [79,80]. An overview of the main genes potentially involved in cancer biology is presented in Figure 1.

For example, genes like WEE1, MYC, and cyclin D1 show circadian regulation and are linked to cancer, as the disruption of their expression can promote tumorigenesis by altering key cell cycle transitions, such as the G0/G1 or G1/S phases [81,82,83]. Other molecules like CDKN1A/p21, VEGF, PDGF receptor, and KIT ligand also exhibit circadian expression patterns and are involved in cellular proliferation and cancer progression [84]. CDKN1A/p21 can regulate cell cycle arrest, but its dysregulation is observed in some cancers [85]. Similarly, VEGF and PDGF receptors contribute to tumor growth, angiogenesis, and metastasis [86,87]. KITLG, which activates tyrosine kinase receptors, plays a role in cancer stemness and metastasis [88]. Finally, cell cycle regulation through apoptosis is also related to circadian rhythms; the disruption of circadian timing can impair apoptosis, allowing potentially cancerous cells to survive [89]. Key tumor suppressors like p53, which maintains genomic integrity, are affected by circadian disruption, increasing the risk of cancer [90]. Other genes involved in cell cycle regulation and apoptosis, such as GADD45A and MDM2, also exhibit circadian expression [91]. When dysregulated, these genes contribute to uncontrolled cell growth and resistance to apoptosis, promoting tumorigenesis [92,93].

Another mechanism related to cell cycle regulation that seems to be associated with eating/fasting timing alternation is circadian autophagy, a process induced in response to calorie restriction and starvation that has been observed to improve longevity and suppress cancer development [94,95]. Some studies showed that TRE has been related to the better regulation of circadian autophagy [96,97]. At the molecular level, the mechanistic target of rapamycin (mTOR) and AMP-activated protein kinase (AMPK) pathways, which regulate anabolic and catabolic processes, respectively, are modulated by the timing of food intake: TRE suppresses mTOR activity during fasting periods, promoting autophagy [98]. This process allows healthy cells to enter a self-maintenance mode, conserving resources and repairing damage during nutrient scarcity in contrast to cancer cells, which often exhibit defective autophagic responses or an inability to enter this protective state due to the continuous activation of oncogenic pathways [99].

### 4.2. Metabolic Disruption, Inflammation, Chronotype and Cancer

Feeding signals act as strong zeitgebers (time cues) for peripheral clocks, particularly in the liver, where they regulate genes involved in glucose metabolism, lipid synthesis, and detoxification [100]. The rationale supporting the retrieved observational findings relies on the hypothesis that consuming the majority of daily energy earlier in the day, when insulin sensitivity peaks, enhances glucose regulation and reduces the risk of metabolic disorders. In contrast, late-evening or nocturnal feeding exacerbates postprandial hyperglycemia and diminishes lipid oxidation, amplifying metabolic disturbances. A morning chronotype aligns feeding periods with the active phase of the circadian cycle and fasting periods with the rest phase, thereby optimizing metabolic processes. In contrast, disruptions in eating patterns can significantly shift the circadian expression of metabolic genes in peripheral organs and the alteration of appetite-regulating hormones, leading to an increase in glucose and insulin levels [100,101]. At the molecular level, a morning chronotype and aligned eating practices improve morning fasting glucose and mean daily glucose levels in association with the expression of circadian clock genes, such as BMAL-1, CRY1, and CRY2 [98]. In several human studies, a morning chronotype [102,103,104] and early TRE [105,106] have been shown to enhance metabolic flexibility by promoting diurnal variations in insulin sensitivity, hepatic glycogen storage, and mitochondrial function. Such a hypothesis is also supported by some epidemiological studies conducted on humans reporting substantially better metabolic health [107]. Moreover, preclinical studies have demonstrated that TRE not only mitigates the metabolic consequences of circadian disruption but also reduces tumor growth in animal models of cancer [108,109], also in association with calorie restriction [110]. TRE has been shown to affect gene expression related to circadian rhythm regulation. Clock genes, including CLOCK, BMAL1, PER, and CRY, exhibit altered expression patterns in response to feeding–fasting cycles, reinforcing the bidirectional relationship between nutrient intake and circadian biology [110].

The resulting metabolic benefits of TRE may stem from its ability to reduce the duration of hyperglycemia and hyperinsulinemia, limiting the activation of insulin and insulin-like growth factor (IGF) pathways that are implicated in cancer development [111]. Chronic hyperinsulinemia promotes the availability of IGF-1 by decreasing the expression of IGF-binding proteins, which would otherwise sequester IGF-1, leading to the inhibition of cellular proliferation and promoting apoptosis [112]. The prolonged fasting period (without an actual calorie restriction within the 24 h span) may still alter systemic metabolic states, initiating changes in key metabolic and hormonal factors that influence cancer cell survival, proliferation, and treatment sensitivity. Some studies conducted on colorectal cancer [113,114], breast cancer [115], and melanoma [116] specifically investigated the role of fasting in altering metabolic pathways limiting cancer aggressiveness and increasing sensitivity to therapy in animal models.

Circadian rhythms also impact the immune system, which plays a role in cancer development via chronic inflammation, driven by circadian-regulated cytokines [117]. The disruption of circadian rhythms, such as through sleep deprivation or light exposure at night, can lead to chronic low-grade inflammation, upregulating pro-inflammatory cytokines like IL-6, IL-1β, and IL-17 [118]. This inflammatory response is linked to genetic changes in clock genes and endocrine disorders, which alter the timing of cytokine release [119]. Inflammation can stimulate tumor growth by promoting cell proliferation and angiogenesis, creating a favorable environment for cancer [120]. A reduction of inflammatory biomarkers has also been observed in individuals adopting TRE or prolonged nighttime fasting [121]. Uncontrolled eating has been associated with increased levels of pro-inflammatory cytokines such as IL-6 and tumor necrosis factor-alpha (TNF-α), which can induce metabolic impairment and promote tumorigenesis by inducing DNA damage and supporting a pro-tumorigenic microenvironment [122]. Also, the reduction of systemic inflammation and improvement in mitochondrial function leads to the enhancement of cellular resilience against oncogenic insults [123].

### 4.3. Circadian Rhythm, Chronotype and Gut Microbiota

Also, it is relevant to highlight that dietary patterns and timing, also known as chrononutrition, are closely related to the microbiome, affecting its composition and function. The intestinal epithelium, which consists of various cell types including enterocytes, goblet cells, and Paneth cells, serves as a critical barrier and defense mechanism against pathogens and harmful substances while also being central to metabolic homeostasis. The gut is home to a significant portion of the body’s immune cells and interacts with the immune system, aiding in pathogen defense and modulating chronic low-grade inflammation. Disruptions to this balance, such as those caused by genetic factors, diet, antibiotics, or circadian rhythm disturbances, can lead to the translocation of harmful substances like lipopolysaccharides, triggering inflammatory responses and compromising gut barrier integrity. Circadian rhythms play a key role in microbiome fluctuations, with microbial diversity and abundance shifting throughout the day in response to feeding and fasting cycles [124]: the circadian regulation of gut functions, including motility and hormone secretion, is tightly linked to the action of clock genes such as CLOCK and Per1. These circadian processes optimize nutrient absorption and digestion. The gut microbiota exhibit diurnal changes influenced by feeding times, aligning with the host’s circadian clock. Disruptions in these rhythms, such as irregular eating patterns, can lead to microbiome imbalances, adversely affecting glucose regulation [125,126]. Studies have revealed that more than half of the total microbial composition fluctuates rhythmically throughout the day, emphasizing the significance of meal timing on gut microbiota composition and function [127]. Furthermore, as reported by several authors, the dominant phyla of the gut microbiota, Firmicutes and Bacteroidetes, exhibit diurnal rhythms, with Firmicutes peaking during feeding and Bacteroidetes during fasting [128,129]. This synchronization between circadian genes and gut microbiota rhythmicity provides evidence of a microbiome-dependent mechanism underlying metabolic disturbances, particularly common in individuals with irregular feeding schedules (such as frequent flyers and shift workers) [130]. Gut microbiota are involved in a variety of processes that can be linked to human’s health. Aside from the transformation and absorption of nutrients and phytochemicals, the gut microbiota impact the host’s epigenetic landscape through the production of metabolites such as short-chain fatty acids (SCFAs) and methyl donors. SCFAs, particularly butyrate, are involved in histone modification, inhibiting histone deacetylases and promoting chromatin remodeling, which in turn influences gene expression [131]. Gut-microbiota-derived metabolites like folate and B vitamins facilitate DNA methylation, contributing to epigenetic regulation. Studies have shown that the gut microbiota induce DNA hypomethylation and enhance the expression of anti-inflammatory and antimicrobial genes, supporting metabolic homeostasis [132]. Furthermore, gut-microbiota-induced epigenetic changes extend beyond the gut, influencing immune cells such as macrophages and dendritic cells, which modulate immune responses. This indicates that gut-microbiota-mediated epigenetic regulation also plays a role in the broader immune system, including gene expression in organs, such as the thymus and spleen. These findings suggest that circadian and immune functions, influenced by both the gut microbiota and epigenetic mechanisms, are integral to maintaining host health and may offer potential therapeutic targets for precision nutrition and disease management.

Some studies have demonstrated that meal timing coordinates the oscillation of gut microbiota and their metabolites, which influence circadian gene expression and play a crucial role in the metabolic response to food intake [133]. Only recently, gut microbiota oscillations have also been associated with chronotype. Specifically, metagenomic analyses revealed that two genera, namely Alistipes and Lachnospira, might be different in the early and late chronotypes; within these genera, *Alistipes putredinis* has been shown to be more abundant in the early chronotype (as compared with both the intermediate and late chronotypes), while *Lachnospira pectinoschiza* was more abundant in the late than in the early chronotype [134]. More recent Mendelian randomization studies showed that Family Bacteroidaceae, Genus Parabacteroides, and Genus Bacteroides were causally associated with the morning chronotype [124]. Whether these relations might be translated toward cancer risk remains to be demonstrated yet [135,136].

## 5. Limitations of Current Research

Circadian rhythms play a pivotal role in regulating numerous genes and cellular processes that are critical for maintaining genomic stability and proper cell cycle progression. The disruption of these rhythms can lead to the dysregulation of cell cycle genes, tumor suppressors, and inflammatory pathways, promoting tumorigenesis and cancer progression. A genetic predisposition could determine circadian misalignment and one’s attitude to prefer morning or evening time to consume food, leading to the further impairment of metabolic health and an increased risk of cancer. Understanding the complex interplay between circadian regulation, eating timing and cancer biology offers potential new avenues for therapeutic interventions aimed at restoring normal circadian function in cancer patients. Despite these insights, significant gaps remain in our understanding of the interplay between chrononutrition and cancer. Many studies have focused on associations rather than causation, and the underlying mechanisms linking meal timing, circadian rhythms, and cancer risk are not fully elucidated. Furthermore, research on specific cancers, such as those of the endocrine system, remains limited, and findings from animal models often require validation in human populations.

## 6. Conclusions

In conclusion, the role of chrononutrition in cancer risk represents a compelling avenue for research, with significant implications for public health and clinical practice. By aligning dietary behaviors with circadian rhythms, it may be possible to mitigate the adverse effects of circadian disruption and support metabolic and cellular processes that protect against cancer. Future studies should aim to integrate dietary interventions with circadian biology to explore how specific eating patterns can modulate cancer risk and progression. This includes investigating the optimal timing, frequency, and composition of meals, as well as the role of chrononutrition in personalized cancer prevention and treatment strategies. Through a deeper understanding of how meal timing and circadian rhythms intersect, researchers and clinicians can develop innovative strategies to reduce cancer burden and promote overall health.

## Figures and Tables

**Table 1:** Main characteristics of observational studies reporting on chronotype and cancer.

Author, Year	Cohort Name, Country	Cancer Type	Study Design	No. Samples, Cases and Controls	Sex, Age	Exposure	Main Findings
Von Behren, 2021, [39]	California Teachers Study, USA	Endometrial cancer	Nested case–control	437 cases, 26,753 controls	F, <90 y	Chronotype measured through a single question based on Horne–Ostberg Morningness–Eveningness Questionnaire	Women who were definite evening types had a statistically significantly increased odds of having cancer (OR = 1.44, 95% CI 1.09–1.91) when compared with morning types.
Costas, 2022, [40]	Screenwide Study, Spain	Endometrial cancer	Hospital-based case–control	180 cases, 218 controls	F, NR	Chronotype assessed through the Munich Chronotype Questionnaire (MCTQ), and through self-reported questions	Inconsistent patterns for chronotype and endometrial cancer risk were observed.
Hurley, 2019, [41]	California Teachers Study, USA	Breast cancer	Nested case–control	2719 cases, 36,967 controls	F, <90 y	Chronotype measured through a single question based on Horne–Ostberg Morningness–Eveningness Questionnaire	Evening chronotype women had a higher likelihood of having cancer (OR = 1.20, 95% CI: 1.06–1.35) compared with morning chronotype women.
Von Behren, 2024, [42]	California Teachers Study, USA	Breast cancer	Prospective cohort	39,555, 1085 cases	F, <90 y	Chronotype measured through a single question based on Horne–Ostberg Morningness–Eveningness Questionnaire	The evening chronotype was associated with an increased risk of breast cancer (HR = 1.19, 95% CI: 1.04–1.36).
McNeil, 2019, [43]	Alberta’s Tomorrow Project, Canada	Various cancer sites	Prospective cohort	19,822, 1322 cases	M and F, 35–69 y	Chronotypes assessed through questions about sleep (habitual sleep duration, bed and wake times, sleep timing midpoint)	A later sleep timing midpoint (indicative of an evening chronotype) was associated with an increased incidence of combined (HR = 1.20, 95% CI: 1.04–1.37) and breast (HR = 1.49, 95% CI: 1.09–2.03) cancers.
Wu, 2022, [44]	UK Biobank, UK	Breast cancer, prostate cancer	Prospective cohort	216,702, 2367 breast cancer cases, 2866 prostate cancer cases	M and F, 40–69 y	Chronotype was self-reported based on touchscreen questionnaire	The evening chronotype was associated with breast cancer risk in females who carry the rs10830963 G risk allele in the melatonin receptor 1B (P trend = 0.015). No association was found for prostate cancer.
Ramin, 2013, [45]	Nurses’ Health Study II, USA	Breast cancer	Prospective cohort	72,517, 1834 cases	F, 15–42 y	Chronotype measured through a specific question and data about night shifts	Participants who self-reported as neither morning nor evening type had a 27% increased risk of breast cancer (OR = 1.27, 95% CI: 1.04–1.56) compared with definite morning types, while none of the other chronotypes were significantly associated with breast cancer risk.
Hansen, 2012, [46]	NA, Denmark	Breast cancer	Nested case–control	219 cases, 899 age-matched controls	F, <75 y	Chronotype measured through a specific question	Frequent night shift work increased the risk for breast cancer (OR = 1.4, 95% CI: 0.9–2.1), especially in participants self-identified as morning chronotypes (OR = 3.9, 95% CI: 1.6–9.5).
Xie, 2021 [47]	UK Biobank, UK	Lung cancer	Prospective cohort	469,691, 2177 cases	M and F, <90 y	Chronotype was self-reported based on touchscreen questionnaire	An evening preference was associated with an elevated lung cancer risk compared with a morning preference (HR = 1.25, 95% CI: 1.07–1.46).
Peeri, 2022, [48]	UK Biobank, UK	Lung cancer	Prospective cohort	382,966, 3644 cases	M and F, <90 y	Chronotype was self-reported based on touchscreen questionnaire	Individuals with slight and definite evening chronotypes were at a greater risk of lung cancer compared with definite morning chronotypes (HR = 1.17, 95% CI, 1.06–1.28 and HR = 1.37, 95% CI, 1.21–1.54, respectively).
Cordina-Duverger, 2022, [49]	Women Epidemiology Lung Cancer Study, France	Lung cancer	Population-based case–control	716 cases, 758 controls	F, 18–75 y	Chronotype measured through a specific question	No association between chronotype and lung cancer was found.
Dickerman, 2016, [50]	Older Finnish Twin Cohort, Finland	Prostate cancer	Prospective cohort	11,370, 602 cases	M, 69.9 ± 8.9 y	Chronotype measured through a questionnaire	Individuals showed that “somewhat evening” types had a significantly increased risk of prostate cancer (HR = 1.3, 95% CI: 1.1–1.6) compared with “definite morning” types.
Cordina-Duverger, 2022, [51]	EPICAP Study, France	Prostate cancer	Population-based case–control	819 cases, 879 controls	M, <75 y	Chronotype was assessed using the Morningness–Eveningness Questionnaire	Men with an evening chronotype showed an increased prostate cancer risk (OR = 1.96, 95% CI: 1.04–3.70).
Lv, 2022, [52]	UK Biobank, UK	Prostate cancer	Prospective cohort	213,999, 6747 cases	M, <90 y	Chronotype was self-reported based on touchscreen questionnaire	Chronotype was not associated with cancer risk.
Lozano-Lorca, 2020, [53]	CAPLIFE Study, Spain	Prostate cancer	Population-based case–control	465 cases, 410 controls	M, 40–80 y	Chronotype was evaluated using the Munich ChronoType Questionnaire (MCTQ) at 40 years old	Night shift work was associated with prostate cancer (OR = 1.47, 95% CI: 1.02–2.11), especially for rotating night shifts (OR = 1.73, 95% CI: 1.09–2.75). The magnitude of the association for ever night work and prostate cancer was higher in evening subjects (OR = 3.14, 95% CI:0.91–10.76) than in morning chronotypes (OR = 1.25, 95% CI: 0.78–2.00).
Papantoniou, 2015, [54]	MCC-Spain Study, Spain	Prostate cancer	Population-based case–control	1095 cases, 1318 controls	M. 27–85 y	Chronotype was estimated as the mid-sleep time on free days corrected for oversleep on free days compared with working days.	Subjects with longer durations of shift work reported a high risk of tumors (≥28 years: RRR 1.63, 95% CI: 1.08–2.45; p-trend = 0.027), especially those with an evening chronotype; tumor risk also increased in morning chronotypes after long-term night work.
Wang, 2023, [55]	UK Biobank, UK	Esophageal cancer	Prospective cohort	393,114, 389 cases	M and F, 37–73 y	Chronotype was self-reported based on touchscreen questionnaire	The evening chronotype was associated with an elevated risk of cancer diagnosed after 2 years of enrollment (HR = 2.79, 95% CI: 1.32–5.88).
Leung, 2019, [56]	Prevention of Ovarian Cancer in Quebec Study, Canada	Ovarian cancer	Population-based case–control	496 cases, 906 controls	F, 18–79 y	Chronotype measured through a specific question	There was no consistent increase in epithelial ovarian cancer risk with longer durations of shift work. The adjusted analysis for the highest shift work category compared with no shift work was 1.20 (95% CI, 0.89–1.63). This association was stronger among individuals identifying as having a “morning” chronotype (OR = 1.64, 95% CI: 1.01–2.65).
Freeman, 2024, [57]	UK Biobank, UK	Pancreatic cancer	Prospective cohort	475,286, 1079 cases	M and F, <90 y	Chronotype was self-reported based on touchscreen questionnaire	No associations were found for evening chronotype and risk of pancreatic cancer in “definitely” an evening person versus “definitely” a morning person, (HR = 0.99, 95% CI: 0.77–1.29).

Abbreviations: F (female), M (male), NA (not applicable); NR (not reported); y (years).

**Table 2:** Main characteristics of Mendelian randomization studies reporting on chronotype and cancer.

Author, Year	Genetic Information	No. Samples for Genetic Information	Data on Cancer	No. Sample Data on Cancer	Exposure (Genetic Traits)	Main Findings
Tian, 2023, [60]	UK Biobank	379,222	UK Biobank	50,252 cases of total cancer and various subtypes	Chronotype (morning), sleep duration, getting up in the morning, nap during daytime, insomnia, narcolepsy	A positive association between the definite evening chronotype and the incidence risk of overall cancer, breast cancer, lung cancer, endometrial cancer, and ovarian cancer was reported. A causal relationship from the Mendelian randomization analysis showed a protective effect of the definite morning chronotype on the risk of overall cancer (OR = 0.91, 95% CI: 0.85–0.97 per category increase), lung cancer (OR = 0.34, 95%CI: 0.26–0.44 per category increase), and breast cancer (OR = 0.69, 95% CI: 0.59–0.80 per category increase), as well as slightly weaker effects for ovarian cancer (OR = 0.61, 95% CI: 0.39–0.97 per category increase) and endometrial cancer (OR = 0.62, 95%CI 0.43–0.91 per category increase), with positive associations between the definite evening chronotype and these types of cancers.
Feng, 2024, [61]	UK Biobank and 23andMe	177,604 and 248,094	Breast Cancer Association Consortium (BCAC)	133,384 cases of breast cancer and 113,789 controls	Chronotype (definite morning, more morning, more evening, definite evening)	The study reported that daytime dozing and the genetically determined morning chronotype are causally linked to a lower risk of breast cancer.
Wu, 2024, [62]	UK Biobank	449,734	Breast Cancer Association Consortium (BCAC) and Discovery, Biology and Risk of Inherited Variants in Breast Cancer Consortium (DRIVE)	122,977 cases of breast cancer and 105,974 controls	Chronotype	A significantly reduced risk of overall breast cancer (OR = 0.89, 95% CI: 0.83–0.94) for the genetically predicted morning chronotype was observed.
Yuan, 2023, [63]	UK Biobank and 23andMe	449,734 and 248,098	UK Biobank and FinnGen	11,952 cases of digestive tract cancer (1339 esophageal, 1086 stomach, 503 liver, 656 biliary tract, 1414 pancreatic and 7543 colorectal cancer) from UK Biobank and 7638 cases of digestive tract cancers (358 esophageal, 889 stomach, 442 liver, 157 biliary tract, 881 pancreatic and 4401 colorectal) from FinnGen	Chronotype	Genetic liability to the morning chronotype was associated with a reduced risk of overall digestive tract cancer (OR = 0.94, 95% CI: 0.90–0.98), stomach cancer (OR = 0.84, 95% CI: 0.73–0.97) and colorectal cancer (OR = 0.92, 95% CI: 0.87–0.98).
He, 2024, [64]	UK Biobank	337,000	UK Biobank	5657 cases of colorectal cancer, 2226 cases of colon cancer, and 1170 cases of rectum cancer	Chronotype (morning), sleep duration, getting up in the morning, nap during daytime, insomnia, snoring, daytime dozing	The study showed that the morning chronotype was marginally associated with the risk of colon cancer (OR = 1.004, 95% CI: 1.000–1.007).
Zong, 2024, [65]	UK Biobank, FinnGen and 23andMe	Up to 462,400	MRC-IEU and Italian data	989 and 701 cases of thyroid cancer and 217,803 and 499 controls	Chronotype, sleep duration, snoring, sleep disorders, getting up in the morning, sleeplessness/insomnia, nap during day	The results showed a potential causal relation between thyroid cancer and the genetically determined morning chronotype.
Sun, 2021, [66]	UK Biobank and 23andMe	449,734 and 248,098	Prostate Cancer Association Group to Investigate Cancer-Associated Alterations in the Genome Consortium (PRACTICAL)	79,148 cases of prostate cancer and 61,106 controls	Chronotype	Individuals with genetically predicted morningness had a reduced causal risk of prostate cancer (OR = 0.71, 95% CI: 0.54–0.94) compared with the eveningness.
Wang, 2021, [67]	UK Biobank	462,434	International Lung Cancer Consortium (ILCCO)	11,348 cases of lung cancer and 15,861 controls	Chronotype, getting up in the morning, sleep duration, nap during the day, sleeplessness	The results showed that sleeplessness was associated with a higher risk of lung adenocarcinoma, while sleep duration played a protective role in lung cancer, with no significant associations with chronotype per se.

**Table 3:** Main characteristics of studies reporting on chrononutrition and cancer.

Author, Year	Cohort Name, Country	Cancer Type	Study Design	No. Samples, Cases and Controls	Sex, Age	Exposure	Main Findings
Palomar-Cros, 2021, [73]	MCC-Spain Study, Spain	Prostate cancer	Population-based case–control	607 cases, 848 controls	M, age range 20–85 y. Cases 66.0 y (±8.4), controls 65.6 y (±7.0)	Circadian data were assessed through a telephone interview	Nighttime fasting >11 h was associated with a reduced risk of prostate cancer compared with those fasting for ≤11 h (OR = 0.77, 95% 0.54–1.07). Combining a long nighttime fasting and an early breakfast was associated with a lower risk of prostate cancer compared with a short nighttime fasting and a late breakfast (OR = 0.54, 95% CI 0.27–1.04).
Palomar-Cros, 2022, [74]	MCC-Spain Study, Spain	Breast cancer	Population-based case–control	1181 cases, 1326 controls	F, 55.4 y (±11.6) cases, 58.4 y (±12.5) controls	Circadian data were assessed through a telephone interview	In premenopausal women, breakfast time was associated with an 18% increase in breast cancer risk (OR = 1.18, 95% CI: 1.01–1.40). No association was found in postmenopausal women. Nighttime fasting was not related to breast cancer.
D’cunha, 2024, [75]	Living Well after Breast Cancer, Australia and New Zealand	Breast cancer	Randomized controlled trial	159 cases	F, 18–75 y 55 ± 9 y	Chronotype was estimated from self-reported sleep logs recorded consecutively for 7 days	Breast cancer survivors with a late chronotype, compared with early, tended to consume a greater proportion of daily calories after 5 PM (*p* < 0.05), eat after 8 PM (*p* < 0.05), and eat less frequently (≥10% difference).
Srour, 2018, [78]	NutriNet-Santé cohort, France	Breast and prostate cancer	Longitudinal cohort	41,389, 428 breast cancer cases, 179 prostate cancer cases	M and F, 59.2 ± 11.3 y	Three 24 h dietary records	Late eaters (after 9:30 pm) had an increased risk of breast (HR = 1.48 [1.02–2.17], *p* = 0.03) and prostate (HR = 2.20 [1.28–3.78], *p* = 0.004) cancers. No association was observed between cancer risk and number of eating episodes, nighttime fasting duration, time of first eating episode or macronutrient composition of the last eating episode.

Abbreviations: F (female), M (male), NA (not applicable); NR (not reported); y (years).

## Data Availability

Data sharing is not applicable to this article as no data were created or analyzed in this study.

## Abbreviations

The following abbreviations are used in this manuscript:

AMPK	AMP-Activated Protein Kinase
BCAC	Breast Cancer Association Consortium
CDKN1A	Cyclin-Dependent Kinase Inhibitor 1A
GADD45A	Growth Arrest and DNA Damage Inducible Alpha
GWAS	Genome-Wide Association Study
IGF	Insulin-Like Growth Factor
KITLG	KIT Ligand
MCC	Multicase–control Study
MCTQ	Munich Chronotype Questionnaire
MDM2	Mouse Double Minute 2 Homolog
mTOR	Mechanistic Target of Rapamycin
PDGF	Platelet-Derived Growth Factor
PRACTICAL	Prostate Cancer Association Group to Investigate Cancer-Associated Alterations in the Genome
SCFAs	Short-Chain Fatty Acids
TNF-α	Tumor Necrosis Factor-Alpha
TRE	Time-restricted eating
TRF	Time-restricted feeding
VEGF	Vascular Endothelial Growth Factor

## References

[1] Rosenwasser A.M., Turek F.W. (2015). Neurobiology of circadian rhythm regulation. Sleep Med. Clin..

[2] Patke A., Young M.W., Axelrod S. (2020). Molecular mechanisms and physiological importance of circadian rhythms. Nat. Rev. Mol. Cell Biol..

[3] Hastings M.H., Maywood E.S., Brancaccio M. (2018). Generation of circadian rhythms in the suprachiasmatic nucleus. Nat. Rev. Neurosci..

[4] Van Drunen R., Eckel-Mahan K. (2021). Circadian rhythms of the hypothalamus: From function to physiology. Clocks & Sleep.

[5] Logan R.W., McClung C.A. (2019). Rhythms of life: Circadian disruption and brain disorders across the lifespan. Nat. Rev. Neurosci..

[6] Drăgoi C.M., Nicolae A.C., Ungurianu A., Margină D.M., Grădinaru D., Dumitrescu I.-B. (2024). Circadian Rhythms, Chrononutrition, Physical Training, and Redox Homeostasis-Molecular Mechanisms in Human Health. Cells.

[7] Zeng Y., Guo Z., Wu M., Chen F., Chen L. (2024). Circadian rhythm regulates the function of immune cells and participates in the development of tumors. Cell Death Discov..

[8] Godos J., Grosso G., Castellano S., Galvano F., Caraci F., Ferri R. (2021). Association between diet and sleep quality: A systematic review. Sleep Med. Rev..

[9] Quarta S., Siculella L., Levante A., Carluccio M.A., Calabriso N., Scoditti E., Damiano F., Lecciso F., Pinto P., García-Conesa M.-T. (2023). Association between Mediterranean lifestyle and perception of well-being and distress in a sample population of university Italian students. Int. J. Food Sci. Nutr..

[10] Godos J., Lanza G., Ferri R., Caraci F., Cano S.S., Elio I., Micek A., Castellano S., Grosso G. (2024). Relation between dietary inflammatory potential and sleep features: Systematic review of observational studies. Med. J. Nutrition Metab..

[11] Sciacca S., Lo Giudice A., Asmundo M.G., Cimino S., Alshatwi A.A., Morgia G., Ferro M., Russo G.I. (2023). Adherence to the Mediterranean diet and prostate cancer severity. Med. J. Nutrition Metab..

[12] Flanagan A., Bechtold D.A., Pot G.K., Johnston J.D. (2021). Chrono-nutrition: From molecular and neuronal mechanisms to human epidemiology and timed feeding patterns. J. Neurochem..

[13] Ahluwalia M.K. (2022). Chrononutrition-When We Eat Is of the Essence in Tackling Obesity. Nutrients.

[14] Kalmbach D.A., Schneider L.D., Cheung J., Bertrand S.J., Kariharan T., Pack A.I., Gehrman P.R. (2017). Genetic basis of chronotype in humans: Insights from three landmark GWAS. Sleep.

[15] Mohawk J.A., Green C.B., Takahashi J.S. (2012). Central and peripheral circadian clocks in mammals. Annu. Rev. Neurosci..

[16] Lotti S., Pagliai G., Colombini B., Sofi F., Dinu M. (2022). Chronotype Differences in Energy Intake, Cardiometabolic Risk Parameters, Cancer, and Depression: A Systematic Review with Meta-Analysis of Observational Studies. Adv. Nutr..

[17] Bell-Pedersen D., Cassone V.M., Earnest D.J., Golden S.S., Hardin P.E., Thomas T.L., Zoran M.J. (2005). Circadian rhythms from multiple oscillators: Lessons from diverse organisms. Nat. Rev. Genet..

[18] Jagannath A., Taylor L., Wakaf Z., Vasudevan S.R., Foster R.G. (2017). The genetics of circadian rhythms, sleep and health. Hum. Mol. Genet..

[19] Cox K.H., Takahashi J.S. (2019). Circadian clock genes and the transcriptional architecture of the clock mechanism. J. Mol. Endocrinol..

[20] Montaruli A., Castelli L., Mulè A., Scurati R., Esposito F., Galasso L., Roveda E. (2021). Biological rhythm and chronotype: New perspectives in health. Biomolecules.

[21] Almoosawi S., Vingeliene S., Gachon F., Voortman T., Palla L., Johnston J.D., Van Dam R.M., Darimont C., Karagounis L.G. (2019). Chronotype: Implications for Epidemiologic Studies on Chrono-Nutrition and Cardiometabolic Health. Adv. Nutr..

[22] Taylor B.J., Hasler B.P. (2018). Chronotype and mental health: Recent advances. Curr. Psychiatry Rep..

[23] Ribas-Latre A., Eckel-Mahan K. (2016). Interdependence of nutrient metabolism and the circadian clock system: Importance for metabolic health. Mol. Metab..

[24] Chauhan S., Norbury R., Faßbender K.C., Ettinger U., Kumari V. (2023). Beyond sleep: A multidimensional model of chronotype. Neurosci. Biobehav. Rev..

[25] de Oliveira Melo N.C., Cuevas-Sierra A., Souto V.F., Martínez J.A. (2024). Biological Rhythms, Chrono-Nutrition, and Gut Microbiota: Epigenomics Insights for Precision Nutrition and Metabolic Health. Biomolecules.

[26] Chaix A., Manoogian E.N.C., Melkani G.C., Panda S. (2019). Time-Restricted Eating to Prevent and Manage Chronic Metabolic Diseases. Annu. Rev. Nutr..

[27] St-Onge M.-P., Mikic A., Pietrolungo C.E. (2016). Effects of diet on sleep quality. Adv. Nutr..

[28] Binks H., E Vincent G., Gupta C., Irwin C., Khalesi S. (2020). Effects of diet on sleep: A narrative review. Nutrients.

[29] Chaput J.-P. (2014). Sleep patterns, diet quality and energy balance. Physiol. Behav..

[30] Dashti H.S., Scheer F.A., Jacques P.F., Lamon-Fava S., Ordovás J.M. (2015). Short sleep duration and dietary intake: Epidemiologic evidence, mechanisms, and health implications. Adv. Nutr..

[31] Hawley J.A., Sassone-Corsi P., Zierath J.R. (2020). Chrono-nutrition for the prevention and treatment of obesity and type 2 diabetes: From mice to men. Diabetologia.

[32] Almoosawi S., Vingeliene S., Karagounis L.G., Pot G.K. (2016). Chrono-nutrition: A review of current evidence from observational studies on global trends in time-of-day of energy intake and its association with obesity. Proc. Nutr. Soc..

[33] Adan A., Archer S.N., Hidalgo M.P., Di Milia L., Natale V., Randler C. (2012). Circadian typology: A comprehensive review. Chronobiol. Int..

[34] Wei F., Chen W., Lin X. (2022). Night-shift work, breast cancer incidence, and all-cause mortality: An updated meta-analysis of prospective cohort studies. Sleep Breath..

[35] Rivera-Izquierdo M., Martínez-Ruiz V., Castillo-Ruiz E.M., Manzaneda-Navío M., Pérez-Gómez B., Jiménez-Moleón J.J. (2020). Shift Work and Prostate Cancer: An Updated Systematic Review and Meta-Analysis. Int. J. Environ. Res. Public Health.

[36] Wang G., Wang J.-J., Lin C.-H., Zhou Q., Wang W.-L., Qin T., Li X., Wang Z.-J. (2022). Association of sleep duration, sleep apnea, and shift work with risk of colorectal neoplasms: A systematic review and meta-analysis. J. Gastrointest. Oncol..

[37] Dun A., Zhao X., Jin X., Wei T., Gao X., Wang Y., Hou H. (2020). Association Between Night-Shift Work and Cancer Risk: Updated Systematic Review and Meta-Analysis. Front. Oncol..

[38] Phoi Y.Y., Rogers M., Bonham M.P., Dorrian J., Coates A.M. (2022). A scoping review of chronotype and temporal patterns of eating of adults: Tools used, findings, and future directions. Nutr. Res. Rev..

[39] Von Behren J., Hurley S., Goldberg D., Clague DeHart J., Wang S.S., Reynolds P. (2021). Chronotype and risk of post-menopausal endometrial cancer in the California Teachers Study. Chronobiol. Int..

[40] Costas L., Frias-Gomez J., Benavente Moreno Y., Peremiquel-Trillas P., Carmona Á., de Francisco J., Caño V., Paytubi S., Pelegrina B., Martínez J.M. (2022). Night work, chronotype and risk of endometrial cancer in the Screenwide case-control study. Occup. Environ. Med..

[41] Hurley S., Goldberg D., Von Behren J., Clague DeHart J., Wang S., Reynolds P. (2019). Chronotype and postmenopausal breast cancer risk among women in the California Teachers Study. Chronobiol. Int..

[42] Von Behren J., Goldberg D., Hurley S., Clague DeHart J., Wang S.S., Reynolds P. (2024). Prospective analysis of sleep characteristics, chronotype, and risk of breast cancer in the california teachers study. Cancer Causes Control.

[43] McNeil J., Barberio A.M., Friedenreich C.M., Brenner D.R. (2019). Sleep and cancer incidence in Alberta’s Tomorrow Project cohort. Sleep.

[44] Wu J., Tan X. (2022). The role of MTNR1B polymorphism on circadian rhythm-related cancer: A UK Biobank cohort study. Int. J. Cancer.

[45] Ramin C., Devore E.E., Pierre-Paul J., Duffy J.F., Hankinson S.E., Schernhammer E.S. (2013). Chronotype and breast cancer risk in a cohort of US nurses. Chronobiol. Int..

[46] Hansen J., Lassen C.F. (2012). Nested case-control study of night shift work and breast cancer risk among women in the Danish military. Occup. Environ. Med..

[47] Xie J., Zhu M., Ji M., Fan J., Huang Y., Wei X., Jiang X., Xu J., Yin R., Wang Y. (2021). Relationships between sleep traits and lung cancer risk: A prospective cohort study in UK Biobank. Sleep.

[48] Peeri N.C., Tao M.-H., Demissie S., Nguyen U.-S.D.T. (2022). Sleep duration, chronotype, and insomnia and the risk of lung cancer: United kingdom biobank cohort. Cancer Epidemiol. Biomarkers Prev..

[49] Cordina-Duverger E., Uchai S., Tvardik N., Billmann R., Martin D., Trédaniel J., Wislez M., Blons H., Laurent-Puig P., Antoine M. (2022). Sleep Traits, Night Shift Work and Lung Cancer Risk among Women: Results from a Population-Based Case-Control Study in France (The WELCA Study). Int. J. Environ. Res. Public Health.

[50] Dickerman B.A., Markt S.C., Koskenvuo M., Hublin C., Pukkala E., Mucci L.A., Kaprio J. (2016). Sleep disruption, chronotype, shift work, and prostate cancer risk and mortality: A 30-year prospective cohort study of Finnish twins. Cancer Causes Control.

[51] Cordina-Duverger E., Cénée S., Trétarre B., Rebillard X., Lamy P.-J., Wendeu-Foyet G., Menegaux F. (2022). Sleep Patterns and Risk of Prostate Cancer: A Population-Based Case Control Study in France (EPICAP). Cancer Epidemiol. Biomarkers Prev..

[52] Lv X., Li Y., Li R., Guan X., Li L., Li J., Si S., Ji X., Cao Y., Xue F. (2022). Relationships of sleep traits with prostate cancer risk: A prospective study of 213,999 UK Biobank participants. Prostate.

[53] Lozano-Lorca M., Olmedo-Requena R., Vega-Galindo M.-V., Vázquez-Alonso F., Jiménez-Pacheco A., Salcedo-Bellido I., Sánchez M.-J., Jiménez-Moleón J.-J. (2020). Night shift work, chronotype, sleep duration, and prostate cancer risk: CAPLIFE study. Int. J. Environ. Res. Public Health.

[54] Papantoniou K., Castaño-Vinyals G., Espinosa A., Aragonés N., Pérez-Gómez B., Burgos J., Gómez-Acebo I., Llorca J., Peiró R., Jimenez-Moleón J.J. (2015). Night shift work, chronotype and prostate cancer risk in the MCC-Spain case-control study. Int. J. Cancer.

[55] Wang X., Tian R., Zong X., Jeon M.S., Luo J., Colditz G.A., Wang J.S., Tsilidis K.K., Ju Y.-E.S., Govindan R. (2023). Sleep behaviors, genetic predispositions, and risk of esophageal cancer. Cancer Epidemiol. Biomarkers Prev..

[56] Leung L., Grundy A., Siemiatycki J., Arseneau J., Gilbert L., Gotlieb W.H., Provencher D.M., Aronson K.J., Koushik A. (2019). Shift work patterns, chronotype, and epithelial ovarian cancer risk. Cancer Epidemiol. Biomarkers Prev..

[57] Freeman J.R., Saint-Maurice P.F., Zhang T., Matthews C.E., Stolzenberg-Solomon R.Z. (2024). Sleep and risk of pancreatic cancer in the UK biobank. Cancer Epidemiol. Biomarkers Prev..

[58] Yarmolinsky J., Wade K.H., Richmond R.C., Langdon R.J., Bull C.J., Tilling K.M., Relton C.L., Lewis S.J., Davey Smith G., Martin R.M. (2018). Causal inference in cancer epidemiology: What is the role of mendelian randomization?. Cancer Epidemiol. Biomarkers Prev..

[59] Richmond R.C., Davey Smith G. (2022). Mendelian randomization: Concepts and scope. Cold Spring Harb. Perspect. Med..

[60] Tian S., Huangfu L., Ai S., Zheng J., Shi L., Yan W., Zhu X., Wang Q., Deng J., Bao Y. (2023). Causal relationships between chronotype and risk of multiple cancers by using longitudinal data and Mendelian randomization analysis. Sci. China Life Sci..

[61] Feng J., Wen Y., Zhang Z., Zhang Y. (2024). Sleep traits and breast cancer risk: A two-sample Mendelian randomization study. Sci. Rep..

[62] Wu X., Yang C., Zou Y., Jones S.E., Zhao X., Zhang L., Han Z., Hao Y., Xiao J., Xiao C. (2024). Using human genetics to understand the phenotypic association between chronotype and breast cancer. J. Sleep Res..

[63] Yuan S., Mason A.M., Titova O.E., Vithayathil M., Kar S., Chen J., Li X., Burgess S., Larsson S.C. (2023). Morning chronotype and digestive tract cancers: Mendelian randomization study. Int. J. Cancer.

[64] He F., Yang F., Tang C., Chen D., Xiong J., Zou Y., Zhao D., Peng D., Qian K. (2024). Association between sleep traits and risk of colorectal cancer: A bidirectional Mendelian randomization study. J. Gastrointest. Oncol..

[65] Zong L., Liu G., He H., Huang D. (2024). Causal association of sleep traits with the risk of thyroid cancer: A mendelian randomization study. BMC Cancer.

[66] Sun X., Ye D., Jiang M., Qian Y., Mao Y. (2021). Genetically proxied morning chronotype was associated with a reduced risk of prostate cancer. Sleep.

[67] Wang J., Tang H., Duan Y., Yang S., An J. (2021). Association between Sleep Traits and Lung Cancer: A Mendelian Randomization Study. J. Immunol. Res..

[68] Stringer E.J., Cloke R.W.G., Van der Meer L., Murphy R.A., Macpherson N.A., Lum J.J. (2024). The Clinical Impact of Time-restricted Eating on Cancer: A Systematic Review. Nutr. Rev..

[69] Lu C., Sun H., Huang J., Yin S., Hou W., Zhang J., Wang Y., Xu Y., Xu H. (2017). Long-Term Sleep Duration as a Risk Factor for Breast Cancer: Evidence from a Systematic Review and Dose-Response Meta-Analysis. Biomed Res. Int..

[70] Zhou T., Wang Z., Qiao C., Wang S., Hu S., Wang X., Ma X., Wang D., Li J., Li Z. (2023). Sleep disturbances and the risk of lung cancer: A meta-epidemiological study. BMC Cancer.

[71] Stone C.R., Haig T.R., Fiest K.M., McNeil J., Brenner D.R., Friedenreich C.M. (2019). The association between sleep duration and cancer-specific mortality: A systematic review and meta-analysis. Cancer Causes Control.

[72] Liu R., Wu S., Zhang B., Guo M., Zhang Y. (2020). The association between sleep duration and prostate cancer: A systematic review and meta-analysis. Medicine (Baltimore).

[73] Palomar-Cros A., Espinosa A., Straif K., Pérez-Gómez B., Papantoniou K., Gómez-Acebo I., Molina-Barceló A., Olmedo-Requena R., Alguacil J., Fernández-Tardón G. (2021). The Association of Nighttime Fasting Duration and Prostate Cancer Risk: Results from the Multicase-Control (MCC) Study in Spain. Nutrients.

[74] Palomar-Cros A., Harding B.N., Espinosa A., Papantoniou K., Pérez-Gómez B., Straif K., Ardanaz E., Fernández Villa T., Amiano P., Gómez-Acebo I. (2022). Association of time of breakfast and nighttime fasting duration with breast cancer risk in the multicase-control study in Spain. Front. Nutr..

[75] D’cunha K., Park Y., Leech R.M., Protani M.M., Marquart-Wilson L., Reeves M.M. (2024). Eating frequency, timing of meals, and sleep duration before and after a randomized controlled weight loss trial for breast cancer survivors. J. Cancer Surviv..

[76] Wang Y., Li F., Li X., Wu J., Chen X., Su Y., Qin T., Liu X., Liang L., Ma J. (2024). Breakfast skipping and risk of all-cause, cardiovascular and cancer mortality among adults: A systematic review and meta-analysis of prospective cohort studies. Food Funct..

[77] Wirth M.D., Turner-McGrievy G., Shivappa N., Murphy E.A., Hébert J.R. (2023). Interaction between Meal-timing and Dietary Inflammatory Potential: Association with Cardiometabolic Endpoints in a 3-month Prospective Analysis. J. Nutr..

[78] Srour B., Plancoulaine S., Andreeva V.A., Fassier P., Julia C., Galan P., Hercberg S., Deschasaux M., Latino-Martel P., Touvier M. (2018). Circadian nutritional behaviours and cancer risk: New insights from the NutriNet-santé prospective cohort study: Disclaimers. Int. J. Cancer.

[79] Masri S., Kinouchi K., Sassone-Corsi P. (2015). Circadian clocks, epigenetics, and cancer. Curr. Opin. Oncol..

[80] Soták M., Sumová A., Pácha J. (2014). Cross-talk between the circadian clock and the cell cycle in cancer. Ann. Med..

[81] Ghelli Luserna di Rorà A., Cerchione C., Martinelli G., Simonetti G. (2020). A WEE1 family business: Regulation of mitosis, cancer progression, and therapeutic target. J. Hematol. Oncol..

[82] Dhanasekaran R., Deutzmann A., Mahauad-Fernandez W.D., Hansen A.S., Gouw A.M., Felsher D.W. (2022). The MYC oncogene—the grand orchestrator of cancer growth and immune evasion. Nat. Rev. Clin. Oncol..

[83] Montalto F.I., De Amicis F. (2020). Cyclin D1 in cancer: A molecular connection for cell cycle control, adhesion and invasion in tumor and stroma. Cells.

[84] Zhu Y., Zheng Y., Dai R., Gu X. (2024). Crosstalk between Circadian Rhythm Dysregulation and Tumorigenesis, Tumor Metabolism and Tumor Immune Response. Aging Dis..

[85] Manousakis E., Miralles C.M., Esquerda M.G., Wright R.H.G. (2023). Cdkn1a/p21 in breast cancer: Part of the problem, or part of the solution?. Int. J. Mol. Sci..

[86] Andrae J., Gallini R., Betsholtz C. (2008). Role of platelet-derived growth factors in physiology and medicine. Genes Dev..

[87] Elebiyo T.C., Rotimi D., Evbuomwan I.O., Maimako R.F., Iyobhebhe M., Ojo O.A., Oluba O.M., Adeyemi O.S. (2022). Reassessing vascular endothelial growth factor (VEGF) in anti-angiogenic cancer therapy. Cancer Treat. Res. Commun..

[88] Sheikh E., Tran T., Vranic S., Levy A., Bonfil R.D. (2022). Role and significance of c-KIT receptor tyrosine kinase in cancer: A review. Bosn. J. Basic Med. Sci..

[89] Rana S., Mahmood S. (2010). Circadian rhythm and its role in malignancy. J. Circadian Rhythm..

[90] Lamont E.W., James F.O., Boivin D.B., Cermakian N. (2007). From circadian clock gene expression to pathologies. Sleep Med..

[91] Gotoh T., Vila-Caballer M., Liu J., Schiffhauer S., Finkielstein C.V. (2015). Association of the circadian factor Period 2 to p53 influences p53’s function in DNA-damage signaling. Mol. Biol. Cell.

[92] Palomer X., Salvador J.M., Griñán-Ferré C., Barroso E., Pallàs M., Vázquez-Carrera M. (2024). GADD45A: With or without you. Med. Res. Rev..

[93] Karni-Schmidt O., Lokshin M., Prives C. (2016). The roles of MDM2 and MDMX in cancer. Annu. Rev. Pathol..

[94] Yun C.W., Lee S.H. (2018). The roles of autophagy in cancer. Int. J. Mol. Sci..

[95] Hwangbo D.-S., Lee H.-Y., Abozaid L.S., Min K.-J. (2020). Mechanisms of lifespan regulation by calorie restriction and intermittent fasting in model organisms. Nutrients.

[96] Ulgherait M., Midoun A.M., Park S.J., Gatto J.A., Tener S.J., Siewert J., Klickstein N., Canman J.C., Ja W.W., Shirasu-Hiza M. (2021). Circadian autophagy drives iTRF-mediated longevity. Nature.

[97] Hu X., Peng J., Tang W., Xia Y., Song P. (2023). A circadian rhythm-restricted diet regulates autophagy to improve cognitive function and prolong lifespan. Biosci. Trends.

[98] Jamshed H., Beyl R.A., Della Manna D.L., Yang E.S., Ravussin E., Peterson C.M. (2019). Early Time-Restricted Feeding Improves 24-Hour Glucose Levels and Affects Markers of the Circadian Clock, Aging, and Autophagy in Humans. Nutrients.

[99] Yin Z., Klionsky D.J. (2022). Intermittent time-restricted feeding promotes longevity through circadian autophagy. Autophagy.

[100] Arble D.M., Ramsey K.M., Bass J., Turek F.W. (2010). Circadian disruption and metabolic disease: Findings from animal models. Best Pract. Res. Clin. Endocrinol. Metab..

[101] Chaput J.-P., McHill A.W., Cox R.C., Broussard J.L., Dutil C., da Costa B.G.G., Sampasa-Kanyinga H., Wright K.P. (2023). The role of insufficient sleep and circadian misalignment in obesity. Nat. Rev. Endocrinol..

[102] Remchak M.-M.E., Heiston E.M., Ballantyne A., Dotson B.L., Stewart N.R., Spaeth A.M., Malin S.K. (2022). Insulin sensitivity and metabolic flexibility parallel plasma TCA levels in early chronotype with metabolic syndrome. J. Clin. Endocrinol. Metab..

[103] Malin S.K., Remchak M.-M.E., Heiston E.M., Battillo D.J., Gow A.J., Shah A.M., Liu Z. (2024). Intermediate versus morning chronotype has lower vascular insulin sensitivity in adults with obesity. Diabetes Obes. Metab..

[104] Malin S.K., Remchak M.-M.E., Smith A.J., Ragland T.J., Heiston E.M., Cheema U. (2022). Early chronotype with metabolic syndrome favours resting and exercise fat oxidation in relation to insulin-stimulated non-oxidative glucose disposal. Exp. Physiol..

[105] Sutton E.F., Beyl R., Early K.S., Cefalu W.T., Ravussin E., Peterson C.M. (2018). Early Time-Restricted Feeding Improves Insulin Sensitivity, Blood Pressure, and Oxidative Stress Even without Weight Loss in Men with Prediabetes. Cell Metab..

[106] Che T., Yan C., Tian D., Zhang X., Liu X., Wu Z. (2021). Time-restricted feeding improves blood glucose and insulin sensitivity in overweight patients with type 2 diabetes: A randomised controlled trial. Nutr. Metab.

[107] Marinac C.R., Natarajan L., Sears D.D., Gallo L.C., Hartman S.J., Arredondo E., Patterson R.E. (2015). Prolonged Nightly Fasting and Breast Cancer Risk: Findings from NHANES (2009-2010). Cancer Epidemiol. Biomarkers Prev..

[108] Walker W.H., Kaper A.L., Meléndez-Fernández O.H., Bumgarner J.R., Liu J.A., Walton J.C., DeVries A.C., Nelson R.J. (2022). Time-restricted feeding alters the efficiency of mammary tumor growth. Chronobiol. Int..

[109] Turbitt W.J., Orlandella R.M., Gibson J.T., Peterson C.M., Norian L.A. (2020). Therapeutic Time-restricted Feeding Reduces Renal Tumor Bioluminescence in Mice but Fails to Improve Anti-CTLA-4 Efficacy. Anticancer Res..

[110] Lu W., Wang J., Wang C., Wang H., Gao W., Ye S., Shen R. (2024). Anti-Tumor Effect and Mechanism Study of Caloric Restriction, Achieved by Time-Restricted Feeding, in Mice. Cancer Control.

[111] Salvadori G., Mirisola M.G., Longo V.D. (2021). Intermittent and periodic fasting, hormones, and cancer prevention. Cancers.

[112] Fanti M., Longo V.D. (2024). Nutrition, GH/IGF-1 signaling, and cancer. Endocr. Relat. Cancer.

[113] Nenkov M., Ma Y., Gaßler N., Chen Y. (2021). Metabolic reprogramming of colorectal cancer cells and the microenvironment: Implication for therapy. Int. J. Mol. Sci..

[114] Weng M.-L., Chen W.-K., Chen X.-Y., Lu H., Sun Z.-R., Yu Q., Sun P.-F., Xu Y.-J., Zhu M.-M., Jiang N. (2020). Fasting inhibits aerobic glycolysis and proliferation in colorectal cancer via the Fdft1-mediated AKT/mTOR/HIF1α pathway suppression. Nat. Commun..

[115] Salvadori G., Zanardi F., Iannelli F., Lobefaro R., Vernieri C., Longo V.D. (2021). Fasting-mimicking diet blocks triple-negative breast cancer and cancer stem cell escape. Cell Metab..

[116] Cortellino S., Quagliariello V., Delfanti G., Blaževitš O., Chiodoni C., Maurea N., Di Mauro A., Tatangelo F., Pisati F., Shmahala A. (2023). Fasting mimicking diet in mice delays cancer growth and reduces immunotherapy-associated cardiovascular and systemic side effects. Nat. Commun..

[117] Xu H., Huang L., Zhao J., Chen S., Liu J., Li G. (2021). The circadian clock and inflammation: A new insight. Clin. Chim. Acta.

[118] Waggoner S.N. (2020). Circadian rhythms in immunity. Curr. Allergy Asthma Rep..

[119] Nakao A. (2014). Temporal regulation of cytokines by the circadian clock. J. Immunol. Res..

[120] Landskron G., De la Fuente M., Thuwajit P., Thuwajit C., Hermoso M.A. (2014). Chronic inflammation and cytokines in the tumor microenvironment. J. Immunol. Res..

[121] Marinac C.R., Sears D.D., Natarajan L., Gallo L.C., Breen C.I., Patterson R.E. (2015). Frequency and Circadian Timing of Eating May Influence Biomarkers of Inflammation and Insulin Resistance Associated with Breast Cancer Risk. PLoS ONE.

[122] Hatori M., Vollmers C., Zarrinpar A., DiTacchio L., Bushong E.A., Gill S., Leblanc M., Chaix A., Joens M., Fitzpatrick J.A.J. (2012). Time-restricted feeding without reducing caloric intake prevents metabolic diseases in mice fed a high-fat diet. Cell Metab..

[123] Kamp D.W., Shacter E., Weitzman S.A. (2011). Chronic inflammation and cancer: The role of the mitochondria. Oncology.

[124] Gutierrez Lopez D.E., Lashinger L.M., Weinstock G.M., Bray M.S. (2021). Circadian rhythms and the gut microbiome synchronize the host’s metabolic response to diet. Cell Metab..

[125] Lotti S., Dinu M., Colombini B., Amedei A., Sofi F. (2023). Circadian rhythms, gut microbiota, and diet: Possible implications for health. Nutr. Metab. Cardiovasc. Dis..

[126] Bishehsari F., Voigt R.M., Keshavarzian A. (2020). Circadian rhythms and the gut microbiota: From the metabolic syndrome to cancer. Nat. Rev. Endocrinol..

[127] Litichevskiy L., Thaiss C.A. (2022). The oscillating gut microbiome and its effects on host circadian biology. Annu. Rev. Nutr..

[128] Weger B.D., Gobet C., Yeung J., Martin E., Jimenez S., Betrisey B., Foata F., Berger B., Balvay A., Foussier A. (2019). The Mouse Microbiome Is Required for Sex-Specific Diurnal Rhythms of Gene Expression and Metabolism. Cell Metab..

[129] Reitmeier S., Kiessling S., Clavel T., List M., Almeida E.L., Ghosh T.S., Neuhaus K., Grallert H., Linseisen J., Skurk T. (2020). Arrhythmic gut microbiome signatures predict risk of type 2 diabetes. Cell Host Microbe.

[130] Nobs S.P., Tuganbaev T., Elinav E. (2019). Microbiome diurnal rhythmicity and its impact on host physiology and disease risk. EMBO Rep..

[131] Nshanian M., Gruber J.J., Geller B.S., Chleilat F., Lancaster S.M., White S.M., Alexandrova L., Camarillo J.M., Kelleher N.L., Zhao Y. (2025). Short-chain fatty acid metabolites propionate and butyrate are unique epigenetic regulatory elements linking diet, metabolism and gene expression. Nat. Metab..

[132] Woo V., Alenghat T. (2022). Epigenetic regulation by gut microbiota. Gut Microbes.

[133] Jakubowicz D., Matz Y., Landau Z., Rosenblum R.C., Twito O., Wainstein J., Tsameret S. (2024). Interaction Between Early Meals (Big-Breakfast Diet), Clock Gene mRNA Expression, and Gut Microbiome to Regulate Weight Loss and Glucose Metabolism in Obesity and Type 2 Diabetes. Int. J. Mol. Sci..

[134] Carasso S., Fishman B., Lask L.S., Shochat T., Geva-Zatorsky N., Tauber E. (2021). Metagenomic analysis reveals the signature of gut microbiota associated with human chronotypes. FASEB J..

[135] Fernandes M.R., Aggarwal P., Costa R.G.F., Cole A.M., Trinchieri G. (2022). Targeting the gut microbiota for cancer therapy. Nat. Rev. Cancer.

[136] Nakatsu G., Andreeva N., MacDonald M.H., Garrett W.S. (2024). Interactions between diet and gut microbiota in cancer. Nat. Microbiol..

